# The *in Vitro* Antimicrobial Efficacy of PDT against Periodontopathogenic Bacteria

**DOI:** 10.3390/ijms161126027

**Published:** 2015-11-13

**Authors:** Philippe A. Haag, Valerie Steiger-Ronay, Patrick R. Schmidlin

**Affiliations:** 1 Private Practice, Zahnarztpraxis an der Murg, Metzgerstrasse 1, Frauenfeld CH 8500, Switzerland; haag@zahnarztpraxis-murg.ch; 2 Center of Dental Medicine, Clinic of Preventive Dentistry, Periodontology and Cariology, University of Zurich, Plattenstrasse 11, Zürich CH 8032, Switzerland; valerie.steiger@zzm.uzh.ch

**Keywords:** PDT, photodynamic therapy, *in vitro*, periodontitis, antimicrobial activity

## Abstract

Periodontitis, an inflammatory disease, is caused by biofilms with a mixed microbial etiology and involves the progressive destruction of the tooth-supporting tissues. A rising number of studies investigate the clinical potential of photodynamic therapy (PDT) as an adjunct during active therapy. The aim of the present review was to evaluate the available literature for the *in vitro* antimicrobial efficacy of photodynamic therapy focusing on the periodontopathogenic bacteria *Aggregatibacter actinomycetemcomitans*, *Porphyromonas gingivalis* and *Fusobacterium nucleatum*. The focused question was: “Is it possible to decrease (at least 3 log steps or 99.9%) or even eliminate bacterial growth by photodynamic therapy *in vitro* when compared to untreated control groups or control groups treated by placebo?” In general, PDT resulted in a substantial reduction of surviving bacteria. However, not all studies showed the desired reduction or elimination. The ranges of log_10_-reduction were 0.38 (58%) to a complete eradication (100%) for *P. gingivalis*, 0.21 (39%) to 100% for *A. actinomycetemcomitans* and 0.3 (50%) to 100% for *F. nucleatum.* In conclusion, further and particularly more comparable studies are needed to evaluate if PDT can be clinically successful as an adjuvant in periodontal therapy.

## 1. Introduction

Periodontitis is an inflammatory disease caused by biofilms with a mixed microbial etiology and leads to a progressive destruction of the teeth-supporting tissues. The main objective of periodontal treatment is the removal of the supra- and subgingival biofilm from the root surface in order to eliminate the pathogenic bacteria, which initiate and cause the progression of periodontal disease [1]. At present, the most widely used treatment to achieve this goal is the mechanical instrumentation of the root surface, *i.e.*, scaling and root planning (SRP). However, residual calculus is often observed after this treatment, especially in deep pockets and in posterior teeth [2]. Further, some pathogens are able to invade the surrounding soft tissues of the periodontal pocket, and re-colonization of treated sites may occur if intraoral niches remain untreated [3]. Therefore, new treatment approaches, such as the antimicrobial PDT (aPDT) have been proposed for a more efficient elimination of the pathogenic biofilm.

Antimicrobial PDT is considered a non-invasive therapeutic method able to selectively target periodontal pathogens, thus avoiding damage to the host tissues [4]. It involves the localization of a photoactivable agent—the photosensitizer—in a target region prior to activation by light of the appropriate wavelength. Singlet oxygen and free radicals are generated upon illumination, which are cytotoxic to microorganisms and their by-products [5]. Today, for curative applications, the photodynamic effects are used for cancers as an alternative to chemo- or radiotherapy; however, local infections such as those that occur within the oral cavity are also increasingly popular fields of application of the aPDT [6]. In the treatment of periodontitis, the additional benefits of the aPDT to the classical scaling and root planning are not completely clear [7]. Therefore, the aim of this review was to evaluate the available literature for the *in vitro* antimicrobial efficacy of photodynamic therapy against periodontopathogenic bacteria. It was analyzed if it is possible to decrease or even stop bacterial growth under laboratory conditions compared to non-treated controls or compared to control groups treated by placebo.

## 2. Results and Discussion

### 2.1. Search and Screening

**Figure 1:**
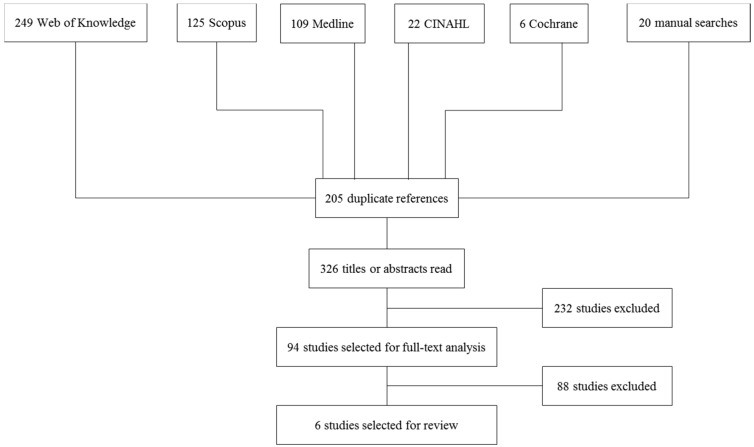
Flowchart of selection process of included studies.

A total of 306 titles from the electronic databases and 20 titles from the hand search were identified (Figure 1). The full texts of 94 publications were further analyzed. Full text analysis led to exclusion of further 25 studies. Only 6 of the remaining 69 papers complied with the inclusion criteria.

### 2.2. Description of Studies

Five out of the six included studies used methylene blue as a photosensitizer [8,9,10,11,12], one applied toluidine blue [13]. The effect of PDT on *Aggregatibacter actinomycetemcomitans* only was investigated in two studies [11,12], *Porphyromonas gingivalis* only was studied in one trial [8]. Two studies investigated three pathogens, *i.e.*, *Aggregatibacter actinomycetemcomitans*, *Porphyromonas gingivalis* and *Fusobacterium nucleatum* [9,10]. One study investigated *Aggregatibacter actinomycetemcomitans* and *Porphyromonas gingivalis* [13].

### 2.3. Antimicrobial Effects of Photodynamic Therapy (PDT) on Porphyromonas Gingivalis

The summarized data set regarding *P. gingivalis* is presented in Table 1.

Chan and Lai [9] performed experiments with methylene blue (MB) as a dye and three different light sources, *i.e.*, a He–Ne laser (632.8 nm) with a 30 mW power output, a 100 mW diode laser at 665 nm, and a 100 mW diode laser at 830 nm. Incubation with MB alone without light irradiation resulted in a statistically significant decrease of *P. gingivalis* [9]. Light irradiation alone without previous incubation with MB showed statistically significant decrease for both diode lasers [9]. Using a MB and light application, all test arrangements led to a statistically significant decrease of *P. gingivalis* [9]. The most efficient combination was MB 0.01% with the diode laser at 665 nm.

Braham *et al.* [8] found no antimicrobial effect using MB only. MB in combination with light irradiation resulted in a decrease of *P. gingivalis* of (3.8 ± 1.3) log_10_. Another study using a diode laser with a wave length in the range of 650–675 nm and MB as a dye resulted in an eradication of >99.9% of planktonic *P. gingivalis* [10].

The reduction of *P. gingivalis* is summarized in Table 4, given as reduction in %, which ranged from 57.97% [9] to 100% [10], as well as log_10_ reduction, which ranged from 0.38 [9] to 6.8 [10].

### 2.4. Antimicrobial Effects of Photodynamic Therapy (PDT) on Aggregatibacter Actinomycetemcomitans

A summary of the data is shown in Table 2.

Chan and Lai [9] found no effect against *A. actinomycetemcomitans* using MB alone in a concentration of 0.01% without light application. MB in combination with light application resulted in a statistically significant decrease of *A. actinomycetemcomitans* with the most efficient combination being MB 0.01% with a diode laser (665 nm). Light application alone without previous incubation with MB, however, led to a statistically significant decrease of *A. actinomycetemcomitans* regardless of the light source applied (He–Ne laser at 632.8 nm, two different diode laser at 665 nm or at 830 nm) as well.

When studying *A. actinomycetemcomitans* organized in a biofilm, Alvarenga *et al.* [12] could not find a statistically significant effect on the viable count of the bacteria, irrespective of the use of dye only (MB), light activation only (diode laser at 660 nm), or dye in combination with a 60 s light application.

The test arrangement for planktonic growth of Street *et al.* [10] using a diode laser with a wavelength of 670 nm and the photosensitizer MB resulted in eradication of >99.9% of planktonic *A. actinomycetemcomitans*.

Eick *et al.* [13] found that toluidine blue and a diode laser (625–635 nm) were effective in reducing the viability of biofilms of two different strains of *A. actinomycetemcomitans*. Similarly, Goulart *et al.* [11] found that exposure of an *A. actinomycetemcomitans* bacterial lineage to a dental photopolymerizer light source (400–500 nm) led to a decrease in bacterial viability both in planktonic culture and as a cellular aggregate in the presence of MB.

The reduction of *A. actinomycetemcomitans* is again summarized in Table 4, given as reduction in %, which ranged from 15% [11] to 100% [10], as well as log_10_ reduction which ranged from 0.07 [11] to 4.9 [10].

### 2.5. Antimicrobial Effects of Photodynamic Therapy (PDT) on Fusobacterium Nucleatum

Table 3 shows the summarized data set regarding *F. nucleatum*.

Chan and Lai [9] found that treatment with MB alone in the absence of laser irradiation did not cause a significant reduction in the viability of the bacteria. A statistically significant decrease of colony forming units was found if a diode laser at a wavelength of either 665 nm or 830 nm illuminated the probes without previous incubation with MB. If MB and a light source (either a He–Ne laser at 632.8 nm, or a diode laser at 665 nm or at 830 nm) were applied, all of the three combinations showed a statistically significant decrease of CFU regarding *F. nucleatum*, with the most efficient set being MB 0.01% in combination with the diode laser (665 nm). Only 4% of the amount of CFU (*F.n.*) compared to the negative control (no dye, no light) grew on the platelets using this combination.

Street *et al.* [10] showed that it is possible to kill nearly 100% of *F. nucleatum* using MB in combination with a diode laser with a wave length of 650–675 nm. The degree of reduction depended on the growing model used (planktonic growth or biofilm), where PDT proved to be more effective in planktonic growing bacteria.

The reduction of *F. nucleatum* is also presented in Table 4, given as reduction in %, which ranged from 49.6% [9] to 100% [10], as well as log_10_ reduction which ranged from 0.3 [9] to 5.2 [10].

**Table 1:** Antimicrobial effects of photodynamic therapy (PDT) on *P.g.*

Reference	Photo-Sensitizer	PDT			Control 1		Control 2		Control 3	
		Light Source	Bacterial Strain	Mean	Conditions	Mean	Conditions	Mean	Conditions	Mean
**Braham *et al.* [8]**	MB 0.01% at dilution 0.03	Diode laser λ: 670 nm P: 150 mW	ATCC 33277	log_10_ reduction 3.8 ± 1.3 *	*N*/*A*	*N*/*A*	*N*/*A*	*N*/*A*	*photosensitizer only*	no effect
**Chan and Lai [9]**	MB 0.01%	He–Ne laser λ: 632.8 nm P: 30 mW He: 6.4 J/cm^2^	ATCC 33277	Viable count CFU 16 ± 5	*negative control* no photosensitizer no light exposure	Viable count CFU 129 ± 7	*light only*	Viable count CFU 132 ± 8	*photosensitizer only*	Viable count CFU 108 ± 12
		Diode laser λ: 665 nm P: 100 mW He: 21.2 J/cm^2^		Viable count CFU 1 ± 0.3		Viable count CFU 117 ± 8		Viable count CFU 67 ± 8		Viable count CFU 111 ± 13
		Diode laser λ: 830 nm P: 100 mW He: 21.2 J/cm^2^		Viable count CFU 58 ± 6		Viable count CFU 138 ± 8		Viable count CFU 81 ± 6		Viable count CFU 126 ± 4
**Eick *et al.* [13]**	TB 0.1 mg/mL	LED lamp λ: 625–635 nm E: 2 W/cm^2^	ATCC 33277	Viable count CFU (8.17 ± 1.01) log_10_	*negative control* no photosensitizer no light exposure	Viable count CFU (9.73 ± 0.46) log_10_	*light only*	Viable count CFU (9.59 ± 0.64) log_10_	*photosensitizer only*	Viable count CFU (9.08 ± 0.25) log_10_
			M5-1-2	Viable count CFU (2.73 ± 3.16) log_10_		Viable count CFU (9.73 ± 0.65) log_10_		Viable count CFU (9.71 ± 0.48) log_10_		Viable count CFU (5.22 ± 4.03) log_10_
**Street *et al.* [10]**	MB 0.01%	Diode laser λ: 670 nm He: 9.4 J/cm^2^	planktonic ATCC 33277	log_10_ reduction 6.8 ± 0.7	*N*/*A*	*N*/*A*	*N*/*A*	*N*/*A*	*N*/*A*	*N*/*A*
	MB 0.01%	Diode laser λ: 670 nm He: 6 J/cm^2^	biofilm ATCC 33277	log_10_ reduction 4.5 ± 1.2	*N*/*A*	*N*/*A*	*N*/*A*	*N*/*A*	*N*/*A*	*N*/*A*

MB: Methylene Blue; TB: Toluidine Blue; λ: wavelength; P: Power Output; E: Irradiance; He: Radiant exposure; min: Minutes; CFU: colony forming units; *: value taken from diagram; *P.g.*: *Porphyromonas gingivalis*; *Exposure time always 60 s unless otherwise specified.*

**Table 2:** Antimicrobial effects of photodynamic therapy (PDT) on *A.a.*

Reference	Photo-Sensitizer	Light Source	Bacterial Strain	PDT	Control 1		Control 2		Control 3	
				Mean	Conditions	Mean	Conditions	Mean	Conditions	Mean
**Chan and Lai [9]**	MB 0.01%	He-Ne laser λ: 632.8 nm P: 30 mW *He:* 6.4 J/cm^2^	ATCC 29522	Viable count CFU 17 ± 6	*negative control* no photosensitizer no light exposure	Viable count CFU 136 ± 12	*light only*	Viable count CFU 116 ± 5	*photosensitizer only*	Viable count CFU 141 ± 10
		Diode laser λ: 665nm P: 100 mW *He:* 21.2 J/cm^2^		Viable count CFU 6 ± 3		Viable count CFU 132 ± 12		Viable count CFU 88 ± 5		Viable count CFU 137 ± 11
		Diode laser λ: 830 nm P: 100 mW *He:* 21.2 J/cm^2^		Viable count CFU 76 ± 5		Viable count CFU 125 ± 11		Viable count CFU 65 ± 10		Viable count CFU 128 ± 8
**Eick *et al.* [13]**	TB 0.1 g/mL	LED lamp λ: 625–635 nm E: 2 W/cm^2^	Y4	Viable count CFU 7.47 ± 1.24 log_10_	*negative control* no photosensitizer no light exposure	Viable count CFU 9.27 ± 0.14 log_10_	*light only*	Viable count CFU 9.36 ± 0.08 log_10_	*photosensitizer only*	Viable count CFU 8.7 ± 0.41 log_10_
			J7	Viable count CFU 4.11 ± 2.77 log_10_		Viable count CFU 6.77 ± 0.78 log_10_		Viable count CFU 6.86 ± 0.8 log_10_		Viable count CFU 5.73 ± 0.21 log_10_
**Goulart *et al.* [11]**	MB 0.5 µmol/L	Dental photo-polymerizer λ: 400–500 nm E: 350–500 mW/cm^2^ He: 0.65 J/cm^2^	planktonic JP2	30 min incubation 15% cell death	*N*/*A*	*N*/*A*	*N*/*A*	*N*/*A*	*photosensitizer only ≤0.1 µmol*/*L* 10 min incubation 30 min incubation	0% reduction
	MB 1 µmol/L	Dental photo-polymerizer λ: 400–500 nm E: 350–500 mW/cm^2^ He: 0.65 J/cm^2^	planktonic JP2	30 min incubation 25% cell death	*N*/*A*	*N*/*A*	*N*/*A*	*N*/*A*	*photosensitizer only* 10 min incubation 30 min incubation	19% reduction * 25% reduction *
	MB 10 µmol/L	*N*/*A*	planktonic JP2	*N*/*A*	*N*/*A*	*N*/*A*	*N*/*A*	*N*/*A*	*photosensitizer only* 10 min incubation 30 min incubation	23% reduction * 31% reduction *
	MB 20 µmol/L	*N*/*A*		*N*/*A*	*N*/*A*	*N*/*A*	*N*/*A*	*N*/*A*	*photosensitizer only* incubation time unclear	50% reduction
	MB 0.5 µmol/L	Dental photo-polymerizer λ: 400–500 nm E: 350–500 mW/cm^2^ He: 0.65 J/cm^2^	biofilm JP2	30 min incubation 73% reduction of absorbance *	*N*/*A*	*N*/*A*	*N*/*A*	*N*/*A*	*photosensitizer only* 30 min incubation	73% reduction of absorbance *
	MB 1 µmol/L		biofilm JP2	30 min incubation 58% reduction of absorbance *	*N*/*A*	*N*/*A*	*N*/*A*	*N*/*A*	*photosensitizer only* 30 min incubation	60% reduction of absorbance *
**Street *et al.* [10]**	MB 0.01%	Diode laser λ: 670 nm He: 9.4 J/cm^2^	planktonic ATCC 33384	log_10_ reduction 1.9 ± 0.6	*N*/*A*	*N*/*A*	*N*/*A*	*N*/*A*	*N*/*A*	*N*/*A*
		Diode laser λ: 670 nm He: 6 J/cm^2^	biofilm ATCC 43717	log_10_ reduction 4.9 ± 1.4	*N*/*A*	*N*/*A*	*N*/*A*	*N*/*A*	*N*/*A*	*N*/*A*
**Alvarenga *et al.* [12]**	MB 100 µM	L: Diode laser λ: 660 nm P: 100 mW He: 15 J/cm^2^	ATCC 29523	log_10_ reduction 0.3	*negative control* no photosensitizer no light exposure	(cfu/mL) 8.87 ± 0.34 log_10_	*light only*	(cfu/mL) 8.13 ± 0.67	*photosensitizer only*	(cfu/mL) 8.47 ± 0.06

MB: Methylene Blue; TB: Toluidine Blue; λ: wavelength; P: Power Output; E: Irradiance; He: Radiant exposure; min: Minutes; CFU: colony forming units; *: value taken from diagram; *A.a.*: *Aggregatibacter actinomycetemcomitans*; *Exposure time always 60 s unless otherwise specified*.

**Table 3:** Antimicrobial effects of photodynamic therapy (PDT) on *F.n.*

Reference	Photosensitizer	Light Source	Bacterial Strain	PDT	Control 1		Control 2		Control 3	
				Mean	Conditions	Mean	Conditions	Mean	Conditions	Mean
**Chan and Lai [9]**	MB 0.01%	He-Ne laser λ: 632.8 nm; P: 30 mW; *He:* 6.4 J/cm^2^	ATCC 23726	Viable count CFU 19 ± 3	*negative control* no photosensitizer no light exposure	Viable count CFU 117 ± 9	*light only* no photosensitizer	Viable count CFU 113 ± 6	*photosensitizer only* no light	Viable count CFU 112 ± 9
		Diode laser λ: 665 nm; P: 100 mW *He:* 21.2 J/cm^2^		Viable count CFU 4 ± 2		Viable count CFU 106 ± 14		Viable count CFU 65 ± 9		Viable count CFU 96 ± 6
		Diode laser λ: 830 nm; P: 100 mW *He:* 21.2 J/cm^2^		Viable count CFU 61 ± 4		Viable count CFU 121 ± 9		Viable count CFU 67 ± 11		Viable count CFU 117 ± 9
**Street *et al.* [10]**	MB 0.01%	Diode laser λ: 670 nm He: 9.4 J/cm^2^	planktonic ATCC 25586	log_10_ reduction 5.2 ± 0.6	*N*/*A*	*N*/*A*	*N*/*A*	*N*/*A*	*N*/*A*	*N*/*A*
		Diode laser λ: 670 nm He: 6 J/cm^2^	biofilm ATCC 25586	log_10_ reduction 3.4 ± 1.1	*N*/*A*	*N*/*A*	*N*/*A*	*N*/*A*	*N*/*A*	*N*/*A*

MB: Methylene Blue; TB: Toluidine Blue; λ: wavelength; P: Power Output; E: Irradiance; He: Radiant exposure; *F.n.*: *Fusobacterium nucleatum*; *Exposure time always 60 s*.

**Table 4:** Overview of the results (percentage reduction and log reduction) of all included studies.

Reference	Bacteria Investigated		PDT		*P.g.*		*A.a.*		*F.n.*	
			Photosensitizer	Light Source	Reduction in %	Log_10_ Reduction	Reduction in %	Log_10_ Reduction	Reduction in %	Log_10_ Reduction
**Braham *et al.* [8]**	*P.g.*		MB 0.01% at dilution 0.03	Diode laser λ: 670 nm; P: 150 mW	99.98	3.8 *	*N*/*A*	*N*/*A*	*N*/*A*	*N*/*A*
**Chan and Lai [9]**	*P.g. A.a. F.n.*		MB 0.01%	He–Ne laser λ: 632.8 nm; P: 30 mW; *He:* 6.4 J/cm^2^	90.7	1.03	87.5	0.9	83.76	0.79
				Diode laser λ: 665 nm; P: 100 mW; *He:* 21.2 J/cm^2^	99.15	2.07	95.45	1.3	96.23	1.42
				Diode laser λ: 830 nm; P: 100 mW; *He:* 21.2 J/cm^2^	57.97	0.38	39.2	0.21	49.59	0.3
**Eick *et al.* [13]**	*P.g. A.a.*	*ATCC 33277 Y4*	TB 0.1 mg/mL	LED lamp λ: 625-635 nm; E: 2 W/cm^2^	97.25	1.56	98.42	1.8	*N*/*A*	*N*/*A*
		*M5-1-2 J7*		LED lamp λ: 625-635 nm; E: 2 W/cm^2^	99.99	7	99.78	2.66	*N*/*A*	*N*/*A*
**Street *et al.* [10]**	*P.g. A.a. F.n.*	*planktonic*	MB 0.01%	Diode laser λ: 670 nm; He: 9.4 J/cm^2^	100	6.8	98.74	1.9	99.99	5.2
	*P.g. A.a. F.n.*	*biofilm*		Diode laser λ: 670 nm; He: 6 J/cm^2^	100	4.5	100	4.9	99.96	3.4
**Alvarenga *et al.* [12]**	*A.a.*		MB 100 µM	L: Diode laser λ: 660 nm; P: 100 mW; He: 15 J/cm^2^	*N*/*A*	*N*/*A*	50	0.3	*N*/*A*	*N*/*A*
**Goulart *et al.* [11]**	*A.a.*		MB 0.5 µmol/L	Dental photopolymerizer λ: 400–500 nm; E: 350–500 mW/cm^2^ He: 0.65 J/cm^2^	*N*/*A*	*N*/*A*	15	0.07	*N*/*A*	*N*/*A*
			MB 1 µmol/L	Dental photopolymerizer λ: 400–500 nm; E: 350–500 mW/cm^2^ He: 0.65 J/cm^2^	*N*/*A*	*N*/*A*	25	0.12	*N*/*A*	*N*/*A*

MB: Methylene Blue; TB: Toluidine Blue; λ: wavelength; P: Power Output; E: Irradiance; He: Radiant exposure; *: value taken from diagram; *Exposure time always 60 s*.

### 2.6. Discussion

The purpose of the present review was to evaluate the available literature for the *in vitro* antimicrobial efficacy of photodynamic therapy regarding *P. gingivalis*, *A. actinomycetemcomitans*, *F. nucleatum*. In the initial search, many studies with different light sources, photosensitizers and application protocols were found. Only six papers could be found that complied with these. Originally, also studies assessing *C. albicans* were planned to be included in this review. Eight studies, which investigated the effect of PDT against *C. albicans*, were included in the fulltext analysis. Finally, however, none of these studies matched the inclusion criteria. Therefore, the studies concerning *C. albicans* had to be excluded and no results regarding antifungal efficacy could be presented.

Only studies were included, which applied either methylene blue or toluidine blue as photosensitizer. This criterion was chosen because these two dyes rank among the most commonly used photosensitizers for oral antimicrobial PDT [14,15]. Both dyes have been shown to have no toxic effect in the commonly used concentration of 0.01%; furthermore, they present with a high degree of selectivity for damage for gram-positive and gram-negative bacteria, as well as viruses and yeasts [16,17,18].

All papers included showed that the respective dye used in combination with a light source resulted in a substantial reduction of the number of surviving bacteria or of the growing bacteria compared to the control groups. In the result section of this review, the individual outcomes of the PDT are presented, and a conversion from percentage reduction to log reduction and *vice versa* was performed for a better overview and comparison of the results. In general, PDT resulted in a substantial reduction of surviving bacteria. However, not all studies showed the desired reduction or elimination. The reduction of *P. gingivalis*, *A. actinomycetemcomitans* and *F. nucleatum* ranged from 57.8% [9], 15% [11] and 49.6% [9] to 100% [10], respectively. The wide range between the publications can be explained by various factors. One important factor is that PDT is more efficient in killing bacteria in their planktonic phase than in biofilms derived from the same plaque samples [19]. This trend was confirmed by studies included in this review, where PDT was more efficient if bacteria were in a planktonic phase than when they were organized in a biofilm [9,10,11]. Another publication, which was not included in this review, could not prove any effect of PDT in a multispecies biofilm because the matrix-embedded microbial populations in a biofilm were well protected against antimicrobial agents [7]. Similar effects are observed using antibiotics in periodontal treatment. These difficulties depend on more than one factor [20]. Two approaches are considered to explain the reduced antibiotic susceptibility. First, the medicament is not able to penetrate the biofilm. Furthermore, antibiotics are more effective in killing rapidly growing cells. This is the second approach. Because of nutrient limitation, microorganisms are growing slowly and their antimicrobial susceptibility is reduced [20]. There are antibiotics not able to kill non-growing cells. Probable reasons that PDT is less effective in biofilms compared to planktonic condition are the distinct and protected phenotypes expressed by dental plaque microorganisms once they attached to the tooth [19]. Furthermore, MB has reduced penetration which may result from presence of proteins derived from gingival crevicular fluid and saliva [19]. Other possibilities are that MB and toluidine blue are substrates of multidrug resistance pumps existing in bacteria [21] or that microorganisms organized in a biofilm are able to exist in a slow-growing or starved state [22]. It was shown in some publications, that the degree of photo-inactivation was dependent upon the time of the irradiation [9,12]. One test arrangement showed a statistically significant decrease of bacteria (*P.g*.) incubated by methylene blue only with no light application [9]. Furthermore, the kind of light activation, concentration of photosensitizer and pathogen treated will also influence the results.

*In vitro* test arrangements have led to significant advances in the study of dental biofilm, but their results cannot be imported into clinical situations [23]. *In vitro* models often involve a small number of species of microorganisms, and are performed under laboratory conditions that cannot reproduce the physiologic situation [24]. Several factors such as salivary flow, the capacity of antimicrobial substances to adhere to the teeth or soft tissues, and the interactions of non-cultivable bacteria cannot be reflected in an *in vitro* set-up [25]. Anyhow it is important to test the validity of any medical procedure before applying it in clinic. This review focused on *in vitro* studies investigating the antimicrobial efficacy of photodynamic therapy. Nevertheless, a rising number of clinical studies are available which focus on PDT as an adjunctive therapy modality to periodontal treatment *in vivo.* A systematic review by Sgolastra and co-workers [26] investigated the potential of antimicrobial photodynamic therapy adjunctive to scaling and root planning in patients with chronic periodontitis. A meta-analysis was conducted to evaluate any clinical adjunctive effect of PDT to SRP when compared to SRP alone or in combination with placebo. Fourteen randomized clinical trials, which were published between 2007 and 2012, were included. The results of the latter review indicated, that the adjunctive use of PDT to SRP could provide additional benefits when compared to SRP alone, in terms of probing depth reduction and clinical attachment level gain. However, these clinical improvements, although statistically significant, proved not to be relevant in terms of clinical applicability. Further, they were only observed at the 3-month-follow up, whereas no significant differences were found after 6 months [26]. Another systematic review corroborated these findings and also showed an additional benefit regarding the treatment of chronic periodontitis with regard to probing depth and clinical attachment level gain [27]. However, only four studies were included in this meta-analysis.

In summary, well-controlled *in vitro* studies as well as randomized clinical trials are necessary to determine whether this adjunctive treatment provides significant additional benefits to periodontal therapy.

## 3. Experimental Section

### 3.1. Search Strategy

The electronic databases CINAHL, Cochrane, Medline, Scopus and WoK were searched for studies published up to and including Mai 2015. The search was limited to laboratory (*in vitro*) studies on photodynamic therapy (PDT) that tested the antimicrobial effect to strains of periodontopathogenic bacteria. No language or time restrictions were applied.

The following strategy was applied: ((photodynam* or photocem*) NEAR/3 (therapy or treatment or intervention or effect)) OR (photochemotherapy or phototherapy) OR (photodynam* or photochem), (dent* or oral or periodont*), (periodont* NEAR 3 (disease or pocket)) OR ((attachment or “alveolar bone”) NEAR/3 (loss or level)) OR (periodontitis or (pocket NEAR/3 depth)), (therapy or treatment or intervention), (root NEAR/3 planing) OR ((dental or periodontal) NEAR/3 (scaling or debridement)) OR (calculus NEAR/3 (remov* or debridement)), (disinfection or antimicrobial or anti-microbial or “anti microbial” or bactericidal or bacteriostatic or anti-infective or antiinfective or “anti infective”) OR ((kill* or inactivat* or inhibit* or block* or viability) NEAR/15 bacteria*), and (“*in vitro*” or “*ex vivo*” or experimental or laboratory) OR ((cell* or strain or bacteria or colony) NEAR/10 (grow* or culture* or count* or plate*)). After title and abstract screening, an additional hand search was performed in the reference lists of all reviews and full texts of interest.

### 3.2. Eligibility Criteria for Studies

Studies were only included if they had an *in vitro*-design, if they used methylene blue or toluidine blue as a dye. Furthermore, we decided to include only studies with an irradiation time of 60 seconds, because most of the studies, which were included in the fulltext analysis, used only this irradiation time. Thereby, a better comparability of the results in this review should to be achieved. With regard to the selected microorganisms, only studies were included that investigated the effect of PDT on *Aggregatibacter actinomycetemcomitans*, *Porphyromonas gingivalis* and *Fusobacterium nucleatum*.

### 3.3. Outcome Measures

The main focus of this study was to critically assess the *in vitro* antimicrobial efficacy of photodynamic therapy focusing on the periodontopathogenic bacteria *Aggregatibacter actinomycetemcomitans*, *Porphyromonas gingivalis* and *Fusobacterium nucleatum*. The focused question was: “Is it possible to decrease (at least 3 log steps or 99.9%) or even eliminate bacterial growth by photodynamic therapy *in vitro* when compared to untreated control groups or control groups treated by placebo?”

### 3.4. Study Selection

First, three reviewers (Philippe A. Haag, Valerie Steiger-Ronay, Patrick R. Schmidlin) independently screened all titles and abstracts of the electronic search and assessed them for possible inclusion in the review. Thereafter, all full texts of potentially eligible studies were assessed. Any disagreement concerning inclusion was resolved by discussion. 

### 3.5. Analysis of the Results

Because it was not possible to extract all data out of the original papers, we tried to contact the corresponding authors per E-Mail asking for raw data. Where this was not possible, we took the values from the diagrams. So there may be a certain inaccuracy in the correspondent values. To present the results we created different tables. To analyze the results we converted the outcomes into different values. For each outcome we converted the percentage value to log reduction value or *vice versa*, if possible. The formulas for these conversions are the following:

The formula to convert percent reduction to log reduction is $L=\;-\left(\log10\left(\frac{-P}{100}+1\right)\right)$ where *P* is the percent reduction and *L* is the log reduction. The formula to convert log reduction to percent reduction is $P=\left(1-10^{-L}\right)\;\times \;100$ where *P* is the percent reduction and *L* is the log reduction. In certain cases, we also needed to calculate the log reduction on the basis of existing values for viable microorganisms. The formula for this calculation is $Log\;Reduction=\;\log10\left(\frac{A}{B}\right)$ where *A* is the number of viable microorganisms before treatment and B is the number of viable microorganisms after treatment.

## 4. Conclusions

This review of the literature does not allow drawing any concrete conclusions regarding the efficacy of PDT due to the small number of included studies and the very different test arrangements. Although PDT seems to be a promising option for reducing the quantity of periodontopathogenic bacteria in combination with conventional therapy modalities. It would be desirable to develop methods they are able to get along with thicker, well organized biofilm. Further, particularly more comparable, studies are needed to evaluate if PDT can be clinically successful as an adjuvant in periodontal therapy.
